# Case report: a Chinese girl with dent disease 1 and turner syndrome due to a hemizygous *CLCN5* gene mutation and Isochromosome (Xq)

**DOI:** 10.1186/s12882-020-01827-4

**Published:** 2020-05-11

**Authors:** Yuhong Ye, Jingjing Wang, Xiaofang Quan, Ke Xu, Haidong Fu, Weiyue Gu, Jianhua Mao

**Affiliations:** 1 Department of Nephrology, The Children’s Hospital of Zhejiang University School of Medicine, #57 Zhugan Lane, Hangzhou, 310003 China; 2 Chigene (Beijing) Translational Medical Research Center Co. Ltd., E2 Biomedical Park, #88 Kechuang Sixth Ave, Yizhuang, Beijing, China

**Keywords:** Dent disease 1, *CLCN5*, Turner syndrome, I(X)(q10), LMWP

## Abstract

**Background:**

Female Dent disease 1 patients with low-molecular-weight proteinuria (LMWP) due to *CLCN5* gene mutation were rarely reported, and these cases that the people were also with Turner syndrome (TS) were even hardly documented before.

**Case presentation:**

Here we report a 3-year and 11-month old Chinese girl with short stature who had a karyotype of 46,X,i(X)(q10) and a de novo pathogenic variant in the *CLCN5* gene on the short arm of X chromosome. Laboratory examinations showed that the patient had LMWP, hypercalciuria, hypophosphatemia, delayed bone age, and genital dysplasia.

**Conclusion:**

The combination of i(X)(q10) and *CLCN5* mutation causes the deletion of the wild-type *CLCN5* allele that results in Dent-1 and TS. To the best of our knowledge, this is the first case that a female *CLCN5* mutation hemizygote is diagnosed with Dent-1 and Turner syndrome due to isochromosome X. Also, our case has indicated that the prevalence of the situation may be largely underestimated because of the mild signs of females with Dent-1.

## Background

Dent disease 1 is a rare X-linked recessive tubulopathy that occurs almost exclusively in males, and in the patients, mild structure abnormalities of proximal tubuli may cause kidney dysfunction. Pathogenic variant in the *CLCN5* gene [1] is found in about 60% of Dent patients (Dent-1, MIM # 300009), whereas *OCRL* gene 15% (Dent-2, MIM # 300555). Dent-1 is characterized by LMWP, hypercalciuria, nephrocalcinosis, kidney stones, renal failure, and rickets [2]. LMWP is the most common phenotype in Dent-1 patients and clinically identified by the elevated levels of urinal β-2-microglobulin (β2M), α-1-microglobulin (α1M) and retinol-binding protein (RBP). Asymptomatic LMWP without nephrotic-range proteinuria (NP) may be observed in almost half of Japanese Dent-1 patients [3], which may indicates a racial specificity in the heterogeneity of Dent-1.

TS is one of the most common chromosomal disorders that affects approximately one in every 2500 females [4]. The clinical features of TS largely depend on the involved regions of the X-chromosome and/or the pattern of X-structural abnormalities. Almost half of TS patients have a karyotype of 45, X, who usually manifests as the full TS phenotypes including short stature, ovarian insufficiency, skeletal dysplasia, heart defects, renal structural anomalies, characteristic facial abnormalities, etc. [4–6], whereas the partial X-structural abnormalities like ring (X) or i (Xq), deletions, duplications and complex rearrangements may cause certain TS phenotypes [7, 8]. However, abnormal X chromosomes can usually tolerate structural abnormalities due to preferential inactivation, which explains why TS patients with 45, X karyotypes may have only mild clinical phenotypes [7].

Here we report a 3-year and 11-month old Chinese girl with unexplained proteinuria, who was diagnosed with heterozygous i (Xq) or TS and asymptomatic LMWP due to a “hemizygous” pathogenic variant in the *CLCN5* gene on short arm of X chromosome.

## Case presentation

The patient was admitted because of having abnormal urine test results when she was 3-year and 11-month old. Physical examination showed she had significant short stature of 93 cm in height (− 2 SD) and relatively lighter weight of 13.6 kg. Neither other physical disorder nor dysmorphia detected. Her unrelated parents and elder sister are normal, and there is no family history of renal disorders.

Laboratory tests indicated that she had LMWP, hypercalciuria, hypophosphatemia, and growth hormone deficiency (Table 1). The results for tests parathyroid hormone, erythrocyte sedimentation rate (ESR), coagulation spectrum, ceruloplasmin, blood lead concentration, determination of trace elements, antinuclear antibodies (ANA), thyroid function, and cytokines by flow cytometry were normal. The renal biopsy was not performed.

**Table 1:** Laboratory test results

	Tests	At diagnosis	At 18 months follow up	Normal
Urine	Total protein	715.7 mg/24 h	709 mg/24 h	< 150 mg/24 h
	Total LMWP	1270 mg/L		
	β2M	25.77 mg/L		0–0.3 mg/L
	α1M	284.47 mg/L		< 12 mg/L
	Microalbumin	245.3 mg/L		< 30 mg/L
	IgG	41.8 mg/L		< 10 mg/L
	Transferrin	28.3 mg/L		< 2.4 mg/L
	RBP	13.7 mg/L		< 0.5 mg/L
	Calcium	81.2, 90.6 mg/24 h^a^	93 mg/24 h	100–300 mg/24 h
	Calcium/creatinine ratio	0.40, 0.46^a^	0.37	< 0.14
Blood	Calcium	2.16 mmol/L	2.37 mmol/L	2.2–2.65 mmol/L
	Creatinine	38 μmol/L	43 μmol/L	~ 44–133 μmol/L
	Phosphorus	1.25 mmol/L	1.2 mmol/L	1.29–2.26 mmol/L
	25(OH)D	21.8 nmol/L	18.4 nmol/L	50–125 nmol/L
	BAP	< 200		~ 12.1–42.7
	Peak level of GH	6.4 ng/ml		> 10 ng/ml
	LH	1.03 IU/L		< 0.4 IU/L
	FSH	36.6 IU/L		0.5–3.2 IU/L
	E2	100 pmol/L		< 149.2 pmol/L

*25(OH) D* 25-hydroxy vitamin D, *BAP* Bone-specific alkaline phosphatase, *E2* Estradiol, *FSH* Follicle-stimulating hormone, *GH* Growth hormone, *IgG* Immunoglobulin G, *LH* Lutenizing hormone, *LMWP* Low molecular weight protamine, *RBP* Retinol binding protein, *α1M* α-1-microglobulin, *β2M* β-2-microglobulin

^a^Results for the two tests

Imaging studies: The renal ultrasound showed bilateral normal-sized kidneys for her age, and no evidence of calculus or nephrocalcinosis. The X-ray imaging indicated the delayed bone age, and the ultrasound scan showed the infantile uterus and bilateral absence of the ovaries.

**Fig. 1:**
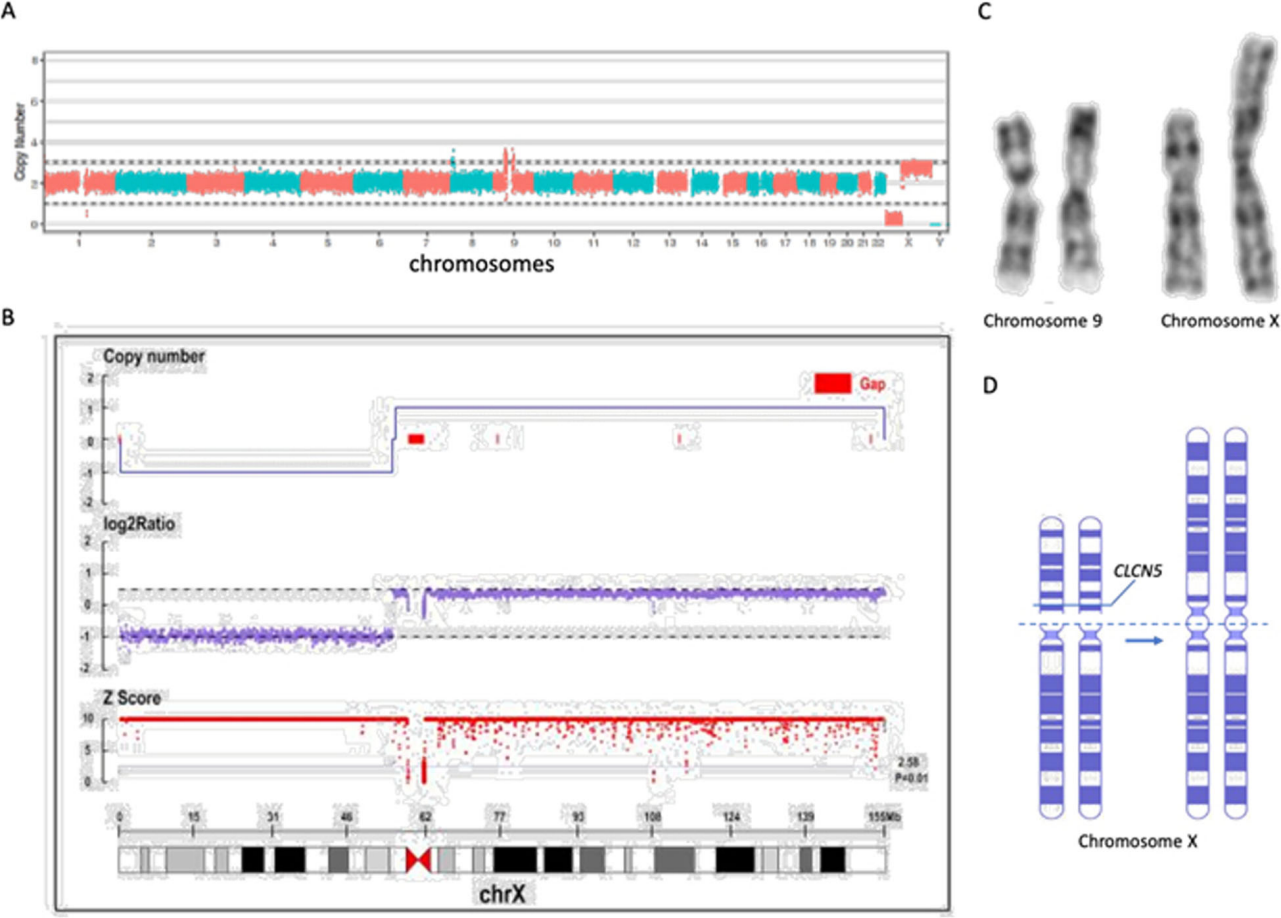
Results of CNV-seq and variant chromosomes. **a** Whole genomic copy number sequencing revealed deletion and duplication of X chromosome. **b** The result of X chromosome copy number shows the heterozygous duplicated long-arm q and the heterozygous deleted short-arm p. **c** Karyotype shows inv(9)(p11q13) pat and i(X)(q10). **d** A schematic representation of the i (Xq), and its cleavage/recombination site on one X chromosome, where the *CLCN5* gene is located on the short arms of X chromosome

**Fig. 2:**
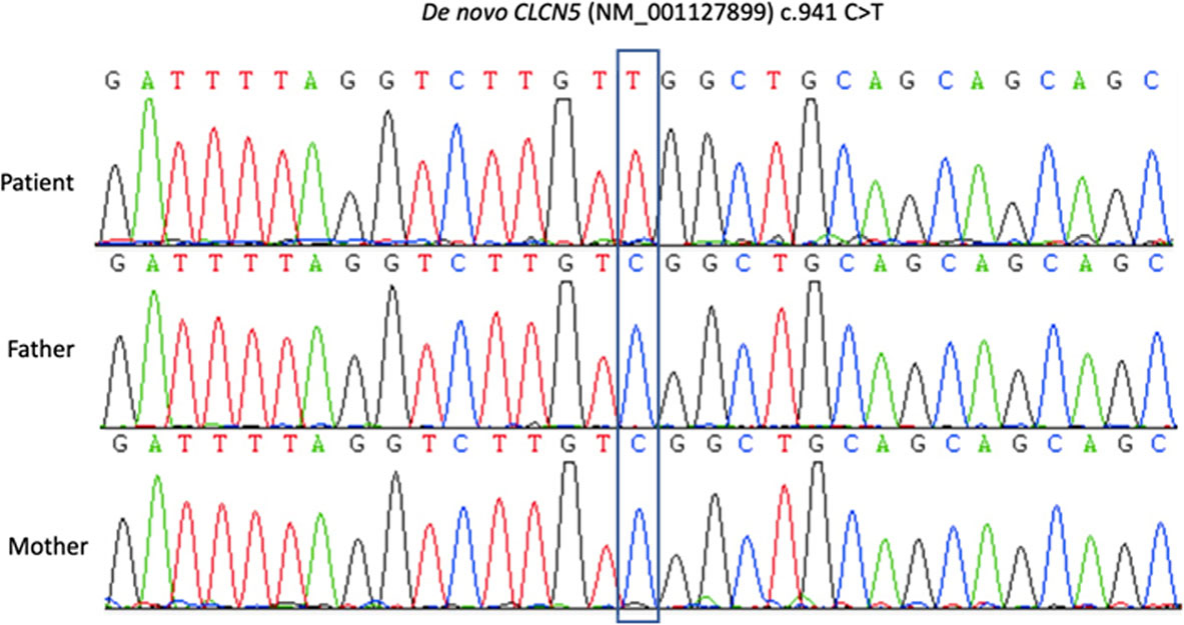
Sanger sequencing confirm the de novo variant NM_000084:c.941C > T

Gene sequencing and Karyotyping: The trio copy number variation sequencing (CNV-seq), a HTS-based method for genome-wide CNV analyzing [9], showed a large de novo CNV on the X chromosome, a heterozygous loss of almost the whole X short arm and a heterozygous duplication of the rest region of the X chromosome (Fig. 1a, b). Karyotyping showed the patient has i(X)(q10) and inv(9)(p11q13) pat (Fig. 1c). Using trio whole exome sequencing (WES), we identified a de novo variant NM_000084:c.941C > T, p.S314L in the *CLCN5* gene on Xp (Fig. 1d), and Sanger sequencing confirmed the variant is de novo and “homozygous” (Fig. 2). The variant S314L is pathogenic according to the ACMG (The American College of Medical Genetics and Genomics) clinical practice guidelines [10]. The defects in *CUBN*, the cubilin-coding gene, was reported the other single-gene cause of asymptomatic LMWP [11], which was excluded by pathogenic or likely pathogenic variant screening in our case. Using HUMARA method [12], X chromosome inactivation (XCI) test showed extremely skewed (98:2) inactivation of the i (Xq) chromosome.

Based on the above results, the patient was diagnosed with Dent disease 1 and Turner Syndrome. In the next 18 months, the child came to the clinic regularly for follow-up, and the condition was stable after examination (Table 1).

## Discussion and conclusions

TS is a canonical knock-out model for studying X chromosome functions, and the most observed karyotype is the classic 45,X (49.2%) followed by 46,X,i (Xq) or i (Xq) mosaic (9.8%) in TS patients [13]. The specificity of phenotypes in TS patients largely depend on the involved regions and the patterns of variations of the X chromosome and the patients with i (Xq) may have specific signs of TS [7, 8]. Therman et al. [14] suggested that loss-of-short-arm X isochromosome is lethal because there is no method of dosage compensation, however, the beneficial effect of X inactivation may result in mild phenotypes in most patients with structural abnormalities of the X chromosome, which is similar to that found in TS patients with 45,X karyotype [7]. The heterogeneity of phenotypes in patients with i (Xq) due to extremely skewed X chromosome inactivation (XCI) are still unclear and, in our patient, short stature and abnormal ovarian development are found.

Isochromosome (Xq) are created by U-type strand exchange, a breakage and reunion in the centric or pericentric region of the p arm, or misdivision occurred in the pericentric region of the p arm, both result in dicentric i (Xq) and loss of the acentric p arm [15]. Loss of the acentric Xp results in a partial monosomy of genes, *CLCN5*, for example, that locate in that portion of the acentric chromosome. (Fig. 1). The *CLCN5*-associated phenotypes may vary, expand from asymptomatic LMWP to severe skeletal dysplasia, and it should be noted that the most Dent-1 patients with asymptomatic proteinuria were reported in Japanese patients [3]. LMWP was defined as proteinuria with urinary proteins less than 40kD [16], which means the patients with isolated LMWP may not have NP. The other patients diagnosed with childhood-onset, isolated LMWP were reported in England [17], which suggests that this condition might not be confine to East Asian patients. Although Shogo Minamikawa et al. [18] demonstrated that the skewed XCI is responsible for some (two of four) of the affected females, it should be noticed that there may be potential heterogeneity of non-X-linked affection in hereditary LMWP patients since some of the fathers (of two of six) had significantly high frequencies of elevated levels of urinary β2M.

Skewed XCI may play a random role in previously reported female Dent-1 cases [18] unless the occurrence can be explained by confirmed findings leading to XCI skewing, as in our patient. Though some studies indicated there may be the X control element (XCE), a cis-elements to regulate XCI, on the X chromosome [19, 20], as previously found in mice [21], there is no confirmed evidence of human XCE, yet. The X chromosome imprinting is a known cause of TS phenotypes, and GH-stimulated heights of patients with imprinted maternal X are significantly shorter than patients with imprinted paternal X [22], which supports the findings in our case. The karyotype of inv(9)(p11q13) pat or other chromosome 9 variants like 9qh+, 9cenh+, 9 ph+, etc., is quite common in general population [23], and we did not find another chromosome 9 linked genetic variation associated with the phenotypes in our patient. Finally, the consistency of asymptomatic LMWP in Japanese and our patient suggests that LMWP is a stand-alone marker for prompting genetic testing in East Asian Dent-1 cases, and moreover, CNV or karyotype, and skewed XCI tests are needed to identify the potential defect of the X chromosome.

In Japanese Dent-1 cases, the patients with LMWP tended to have hypercalciuric nephrocalcinosis [24, 25], but the slow natural course of Dent-1 is a major obstacle to the follow-up study. For the further study, we recommend a large scale of a retrospective study in suspected Dent-1 cases in China, since the prevalence of the disease in China may be largely underestimated because of the mild signs of female patients with Dent-1.

Unexplained LMWP is a sensitive marker for prompt genetic testing in East Asian patients. Our study in this unique, as far as we know, case expanded the spectrum of genotypes, phenotypes and racial specificity of Dent-1, and it raises a new issue to update the criteria for diagnosing Dent-1 disease in Chinese patients because the prevalence of Dent-1 in China might be underestimated.

## Data Availability

The datasets used and/or analyzed during the current study are available from the corresponding author on reasonable request.

## Abbreviations

ACMG: American College of Medical Genetics and Genomics
CNV-seq: Copy number variation sequencing
i (Xq): Isochromosome (Xq)
LMWP: Low-molecular-weight proteinuria
NP: Nephrotic-range proteinuria
RBP: Retinol-binding protein
TS: Turner syndrome
WES: Whole exome sequencing
XCE: X control element
XCI: X chromosome inactivation
XLR: X-linked recessive
α1M: α-1-microglobulin
β2M: β-2-microglobulin
